# Transport of Au(III) from HCl Medium across a Liquid Membrane Using R_3_NH^+^Cl^−^/Toluene Immobilized on a Microporous Hydrophobic Support: Optimization and Modelling

**DOI:** 10.3390/membranes10120432

**Published:** 2020-12-17

**Authors:** Francisco J. Alguacil, Lorena Alcaraz, Olga R. Largo, Félix A. López

**Affiliations:** National Center for Metallurgical Research (CENIM), Spanish National Research Council (CSIC), Avenida Gregorio del Amo 8, 28040 Madrid, Spain; fjalgua@cenim.csic.es (F.J.A.); alcaraz@cenim.csic.es (L.A.); olga.rodriguez@csic.es (O.R.L.)

**Keywords:** membrane transport, A327H^+^Cl^−^ ionic liquid, gold, hydrochloric acid, nanoparticles

## Abstract

By the use of the tertiary amine A327 and 1 M HCl solution as precursors, the ionic liquid A327H^+^Cl^−^ was generated and used to investigate its performance in the transport of Au(III) from hydrochloric acid medium. The influence of the stirring speed (600–1800 min^−1^), ionic liquid concentration (1.25–50% *v/v*) in the membrane phase, and gold concentration (0.01–0.15 g/L) in the feed phase on metal transport have been investigated. An equation which included both equilibrium and kinetics parameters was derived, and the membrane diffusional resistance (Δ_m_) and feed phase diffusional resistance (Δ_f_) was estimated as 9.5 × 10^6^ s/cm and 307 s/cm, respectively. At carrier concentrations in the 5–50% *v/v* range and gold concentrations in the 0.01–0.15 g/L range, metal transport is controlled by diffusion of metal species through the feed boundary layer, whereas at the lowest carrier concentrations, membrane diffusion is predominant. From the receiving solutions, gold can be recovered as gold nanoparticles.

## 1. Introduction

Nowadays the presence of metals in urban environments is a norm, such as the necessity of recycling of the materials contained in them, and thus, the concept of urban mining arises. From all the metal-bearing solid waste produced by mankind, and also from years ago, the recycling of e-waste is an environmental and economic issue [1]. In the treatment of these e-wastes, hydrometallurgy can be an option; this technology included leaching of the product yielded from the corresponding pre-treatment of the solid waste (dismantling, shedding, comminution), and separation and recovery of the valuable elements from the leachate. Among these separation technologies, liquid membranes processes have been of increasing interest (though not a single process has been yet installed in industrial form) against liquid-liquid extraction, or even ion exchange and adsorption processes, for the separation and concentration of metals, from dilute aqueous solutions, due to the fact that they combine in a single operation the extraction and stripping stages. Two types of liquid membrane are considered: supported and unsupported liquid membranes, the former consisting of a thin microporous and hydrophobic polymer support impregnated with the carrier (organic phase containing the extractant), which separates the feed and receiving or stripping solutions. From an engineering and practical point of view, supported liquid membranes are of particular interest due to their stability (if corrected operated) and simplicity.

One of the metals encountered in such e-wastes is gold [2,3,4], in fact, this precious metal is found in dentistry, jewellery, unused equipment, industrial fittings and the mentioned e-scraps mentioned above, being, with a high probability, the recovery of this metal from these wastes more profitable than the recovery of gold from raw materials, i.e., about 200 g/gold can be recovered from 1 ton/circuit boards against 1 g/gold from 1 ton/ore. Very often, the recovery of gold from these wastes is done by leaching of the waste with aqua regia, resulting in a leachate in which gold is present as AuCl_4_^−^ or HAuCl_4_ [5]. The recovery of this precious metal from the HCl solution can be done mainly by activated carbon [6], ion exchange resins [7,8,9,10], liquid-liquid extraction using conventional [11,12,13] and ionic liquid [14,15,16,17] extractants, different adsorbents [18,19,20,21,22], and as mentioned above by liquid membranes [23,24,25,26]; more recently, the use of carbon nanotubes have been also considered in the treatment of diluting Au(III)-bearing HCl solutions [27,28,29]. 

The aim of this investigation is to evaluate parameters to optimize performance of flat-sheet supported liquid membrane for the active transport of gold(III) from HCl medium. The overall mass transfer coefficient (K_Au_) was calculated under various experimental parameters, whereas other mass transfer parameters were determined for the Au(III)-HCl-A327H^+^Cl^−^ system. The treatment of the receiving solutions with sodium borohydride leads to the precipitation of gold as nanoparticles. 

## 2. Materials and Methods

### 2.1. Reagents and Solutions

The precursors for the generation of the ionic liquid were the tertiary amine A327 (Sanofi), which is composed by a 50% mixture of tri-octyl and tri-decyl amines, with average molecular weight of 395 and density 0.82 g/cm^3^ (20 °C), the reagent was diluted in toluene (Fluka, Madrid, Spain) in order to adequately determine the range of amine concentrations, and thus, of the ionic liquid to the gold transport experiments. A 1 g/L gold(III) stock solution was prepared by dissolving HAuCl_4_ (Fluka) with 6 M HCl. All the chemicals used in the experimentation, except the amine, were of A.R. grade. The solid support used in the present work was Millipore Durapore (Darmstadt, Germany) GVHP4700 (polyvinylidene fluoride) of 75% porosity, 1.67 tortuosity and 12.5 × 10^−3^ cm thickness.

### 2.2. Methods

#### 2.2.1. Liquid-Liquid Extraction Experiments

The ionic liquid A327H^+^Cl^−^ was generated by mixing in thermostatted separatory funnels equal volumes of organic solutions of the amine A327 in toluene with 1 M HCl solutions for 5 min at 20 °C. Previous experiments showed that equilibrium was reached within 1 min of contact between both phases. After the quick phase disengagement (less than 30 s), the HCl concentration in the organic phases was determined by titration, in ethanol medium, of the corresponding aliquots with standard NaOH solutions, and using bromothymol blue as indicator. The analytical method has less than ±2% variation of the results.

Gold(III) liquid-liquid extraction experiments were done using the same experimental protocol as above, but mixing for 10 min. Previous experiments showed that maximum gold extraction efficiency was found in the 2–4 M HCl range. Gold(III) was analyzed in the aqueous solutions by atomic absorption spectrometry, being found to be reproducible within ~3%, and the metal concentration in the equilibrated organic solutions was estimated by the corresponding mass balance.

#### 2.2.2. SLM Experiments 

Single-stage permeation experiments were carried out in a two-compartment cell which consisted of a feed solution half-cell (200 cm^3^) separated from the receiving solution half-cell (200 cm^3^) by the solid support having an effective membrane area of 11.3 cm^2^. The feed and the receiving solutions were mechanically stirred, at 20 °C, to avoid concentration polarization conditions at the support interfaces and in the bulk of both solutions.

The solid support used in the present work was Millipore Durapore GVHP4700 (Darmstadt, Germany), formed by a microporous polyvinylidenedifloride film of 12.5 × 10^−3^ thickness, 75% porosity, 1.67 tortuosity, and 0.22 μm effective pore size. The supported liquid membrane was prepared by impregnation of the solid support, with the corresponding organic solution, by immersion for 24 h and then left to drip for 15 s before being placed in the cell.

Metal transport was determined by monitoring gold(III) concentrations in the feed and receiving phases as a function of time by atomic absorption spectrometry. The gold(III) concentration in the solutions was found to be reproducible within ±5%. The overall mass transfer coefficient (K_Au_) was computed using the next equation:

(1) $$\ln\frac{{\left[\mathrm{Au}\right]}_{f,t}}{{\left[\mathrm{Au}\right]}_{f,0}}=-\frac{A_{m}K_{\mathrm{Au}}}{V_{f}}t$$

where A_m_ support area, V_f_ is the volume of the feed solution, [Au]_f,t_ and [Au]_f,0_ are the gold concentrations in the feed solution at an elapsed time and time zero, respectively, and t is the elapsed time.

The percentage of gold transported to the receiving solution was calculated by:

(2) $$\%T=\frac{{\left[\mathrm{Au}\right]}_{r,t}}{{\left[\mathrm{Au}\right]}_{f,0}-{\left[\mathrm{Au}\right]}_{f,t}}100$$

where [Au]_r,t_ is the gold concentration in the receiving solution at an elapsed time.

The precipitation of the Au(III)-bearing thiocyanate solution was done in a glass reactor with gentle stirring (50 min^−1^) and at 20 °C. To the gold solution, a NaHB_4_ solution was dropwise added during 15 min; from the first drop, a dark precipitated was formed with the evolution of hydrogen gas, after, the precipitated was separated from the solution by filtration, washed with water, and was left to dry in a desiccator under CaCl_2_.

The solid obtained had a dark brown-purple color and was observed using an Olympus optical microscope model PMEV (Tokyo, Japan). The morphology and chemical composition were carried out by Field Emission Scanning Electronic Microscopy using a Hitachi S-4800 (Chiyoda, Japan) equipped with an energy dispersive X-ray microanalyzer (EDX) from the Oxford INCA Instrument (EDS, Oxford, Concord, MA, USA).

## 3. Results and Discussion

It is worth to note here that in all the experimentation, the ionic liquid was dissolved in toluene. Though many authors claimed that ionic liquids must be used without dilution, which can be true, in real practice it is better to dissolve in a safe and suitable organic diluent to ensure:

(i) the use of ionic liquid concentrations within the adequate range for the given metal-organic system. This practice avoided the use of an excessive and unusable ionic liquid concentration, which used to be the most expensive item of a process,
(ii) to reduce the organic phase viscosity. This is important because it facilitates the phase separation in liquid-liquid experiments, and also in supported liquid membranes methodology, because in many systems, the increase of the organic phase viscosity tends to increase the membrane resistance to the metal transport.

### 3.1. Preparation of R_3_NH^+^Cl^−^ Ionic Liquid 

**Figure 1:**
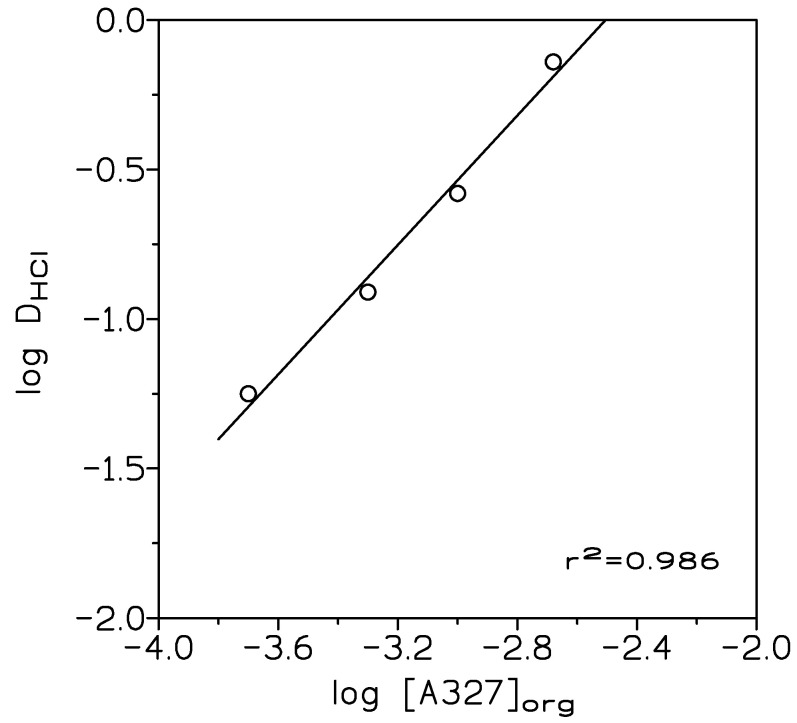
Variation of log D_HCl_ with log [A327]_org_. Aqueous phases: 1 M HCl. Organic phase: 2.5–10% *v/v* (0.05–0.4 M) amine A327 in toluene. Temperature: 20 °C. Time: 10 min. V_org_/V_aq_: 1.

This ionic liquid was prepared by reaction of the tertiary amine diluted in toluene and 1M HCl solutions, and the results were estimated by the distribution coefficient D, defined as:

(3) $$D_{\mathrm{HCl}}=\frac{{\left[\mathrm{HCl}\right]}_{\mathrm{org}}}{{\left[\mathrm{HCl}\right]}_{\mathrm{aq}}}$$

where [HCl]_org_ and [HCl]_aq_ were the HCl concentrations in the extracted phase and in the raffinate or aqueous solution, at the equilibrium, respectively. A plot of log D versus log [A327]_org_ (Figure 1), resulted in a straight line of slope 1.08 (r^2^ = 0.986), thus, the ionic liquid was formed (99.8% amine conversion) accordingly to the equilibrium:

(4) H+aq+Cl+aq−R3Norg⇔R3NH+Clorg−

org and aq subscripts were the extracted phase and the raffinate, respectively. 

To verify the above, the experimental data were treated by a tailored computer program with minimizes the U function, defined as:

(5) $$U=\Sigma {\left(\log D_{\mathrm{cal}}-\log D_{\exp}\right)}^{2}$$

being D_exp_ and D_cal_ the experimental distribution coefficients and the corresponding values calculated by the program. The results indicated that the ionic liquid was formed as indicated in Equation (4), with log K_HCl_ (K_HCl_ being the equilibrium constant related to Equation (4)) 2.65 and U 2.3 × 10^−5^.

### 3.2. Gold(III) Extraction Equilibrium

The extraction of gold(III) by the ionic liquid is based on an anion exchange equilibrium. The gold(III) ions in hydrochloric acid solutions (present as AuCl_4_^−^) form a complex with the extractant R_3_NH^+^Cl^−^ expressed as:

(6) $$R_{3}{\mathrm{NH}}^{+}{\mathrm{Cl}}_{\mathrm{org}}^{-}+\mathrm{Au}{\mathrm{Cl}}_{4_{\mathrm{aq}}}^{-}\Leftrightarrow R_{3}{\mathrm{NH}}^{+}\mathrm{Au}{\mathrm{Cl}}_{4_{\mathrm{org}}}^{-}+{\mathrm{Cl}}_{\mathrm{aq}}^{-}$$

The extraction equilibrium can be described by the next equation:

(7) $$K_{\mathrm{ext}}=\frac{{\left[R_{3}{\mathrm{NH}}^{+}\mathrm{AU}{\mathrm{Cl}}_{4}^{-}\right]}_{\mathrm{org}}{\left[{\mathrm{Cl}}^{-}\right]}_{\mathrm{aq}}}{{\left[R_{3}{\mathrm{NH}}^{+}{\mathrm{Cl}}^{-}\right]}_{\mathrm{org}}{\left[\mathrm{Au}{\mathrm{Cl}}_{4}^{-}\right]}_{\mathrm{aq}}}$$

Using the same computer program than above, it is found that the value of log K_ext_ is found to be 5.99 and U = 0.270.3.3. Gold(III) Transport Across the Supported Liquid Membrane.

**Figure 2:**
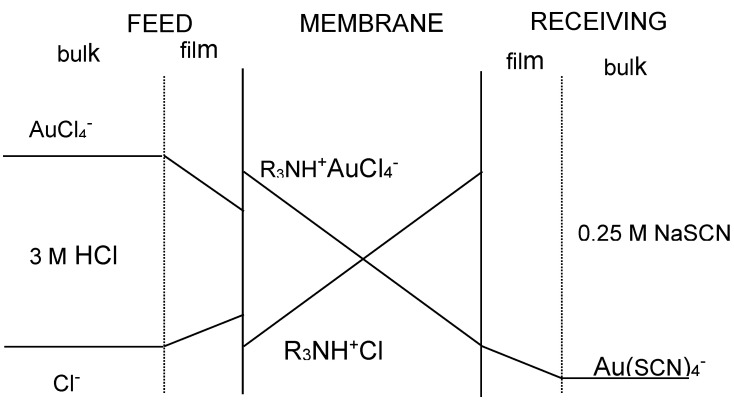
Concentration profile of the species across the supported liquid membrane.

The transport of gold(III) across the membrane containing the ionic liquid phase is described by applying Fick’s first diffusion law to the diffusion layer at the feed phase side, to the membrane phase, and to the receiving phase, though this last contribution is often negligible compared with that at the feed phase side since the distribution coefficient of Au(III) between the membrane and the receiving phases uses to be much lower than the value between the feed and the membrane phases. Figure 2 shows a probable transport scheme for Au(III) with the ionic liquid (R_3_NH^+^Cl^−^) dissolved in toluene through a supported liquid membrane. Accordingly, the transport of Au(III) is associated with a mechanism of co-transport, being the driving force of the difference in acidity between the feed and receiving phases.

In order to yield effective gold(III) transport across the supported liquid membrane, it is of importance to investigate the influence of the stirring speed, applied to the feed phase, on the overall mass transfer coefficient. The transport of gold(III) across the supported liquid membrane is dominated by diffusional resistances which can be of two types: i) the resistance associated with the feed phase boundary layer, and ii) that associated with the membrane support. It is not rare that the magnitude of the first computed with the value of the support resistance [30]. In the present work, stirring of the feed solution was carried out from 600–1500 cm^−1^ (Table 1).

Feed phase: 0.01 g/L Au(III) in 3 M HCl. Stirring speed: variable. Membrane phase: 10% *v/v* ionic liquid in toluene supported on GVHP4700. Receiving phase: 0.25 M NaSCN. Stirring speed: 500 min^−1^. Temperature: 20 °C

The overall mass transfer coefficient increased from 600 to 1000 min^−1^, thus, indicates that there is a continuous decrease of the feed boundary layer thickness with the increase of the stirring speed and that a minimum in this thickness is reached about 1000 min^−1^. The maximum value of the overall mass transfer coefficient is in the same range as that found in other systems using ionic liquids as carriers for gold(III) transport [23,24,25]

In the case of the receiving phase, and if the stirrer in the half-cell is very close to the membrane support, the thickness of the boundary layer is considered to be minimized and the resistance in this side can be neglected [31]. Moreover, the variation of the receiving phase composition from 0.25 to 0.5 M NaSCN has no effect either on gold transport or on the percentage of gold recovered (55% after 3 h) in the receiving solution. In this phase, gold is recovered as Au(SCN)_4_^−^ complex (log β = 43.66) [32] accordingly to the next reaction:

(8) $$R_{3}{\mathrm{NH}}^{+}\mathrm{Au}{\mathrm{Cl}}_{4_{m}}^{-}+4{\mathrm{SCN}}_{r}^{-}\to R_{3}{\mathrm{NH}}^{+}{\mathrm{Cl}}_{m}^{-}+\mathrm{Au}{\left(\mathrm{SCN}\right)}_{4_{r}}^{-}+3{\mathrm{Cl}}_{r}^{-}$$

which also regenerates the ionic liquid. In the above equation, the subscripts m and r denote the membrane and receiving phases, respectively. It should be noted here, that during the transport process the acidity of the receiving phase increases from near neutral to pH 1–2, which reinforced the previous assumption that the co-transport mechanism is the driving force for the present system. 

**Figure 3:**
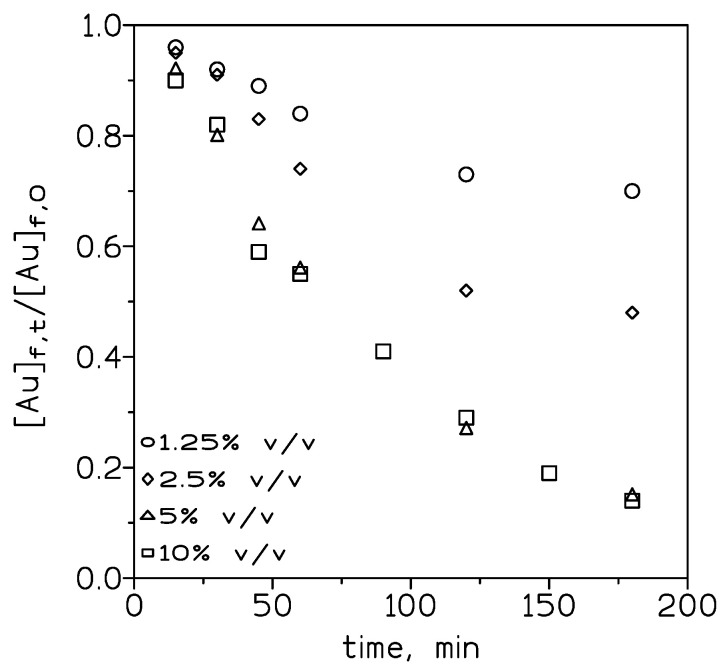
Experimental courses of Au(III) concentration in the feed phase at various ionic liquid concentrations. Feed phase: 0.01 g/L Au(III) in 3 M HCl. Stirring speed: 1000 min^−1^. Membrane phase: ionic liquid in toluene supported on GVHP4700. Receiving phase: 0.25 M NaSCN. Stirring speed: 500 min^−1^. Temperature: 20 °C.

To evaluate the influence of the carrier concentration on the transport of Au(III), various concentrations of the ionic liquid in toluene were employed. Figure 3 shows that in the feed phase, the dimensionless [Au]_f,t_/[Au]_f,0_ relationship decreased when the ionic liquid concentration is increased from 1.25% to 5% *v/v* and then remained constant (5% to 50% *v/v*).

**Figure 4:**
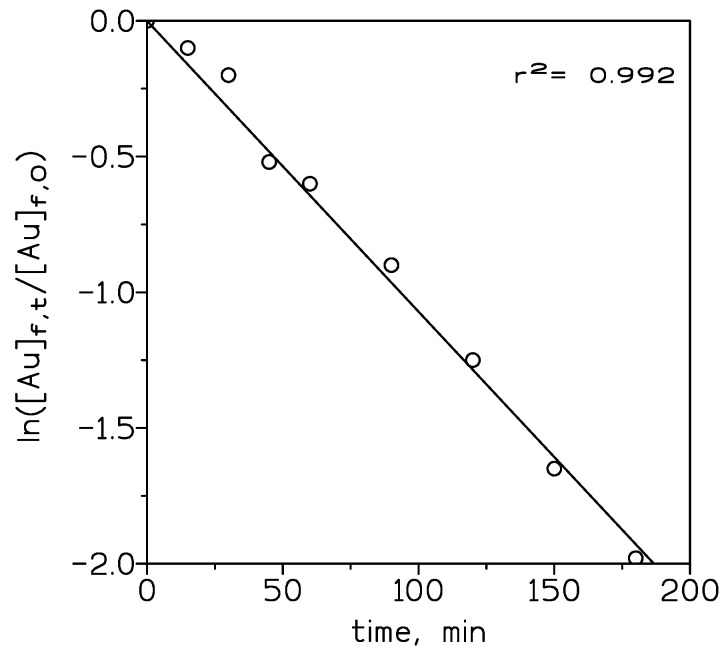
Variation of ln ([Au]_f,t_/[Au]_f,0_ versus time. Organic phase: 10% *v/v* ionic liquid in toluene. Other experimental variables as in Figure 3.

The overall mass transfer values were calculated using equation (1) and 10% *v/v* ionic liquid in toluene, as a representative case for the influence of the concentration of the ionic liquid on mass transfer, and showed in Figure 4. The mass transfer coefficient K_Au_ was increased from 5.9 × 10^−4^ cm/s for ionic liquid (1.25% *v/v*) to 3.1 × 10^−3^ cm/s for ionic liquid (5% *v/v*). At lower concentrations of the ionic liquid, mass transfer control is in the solid support or liquid membrane. Beyond 5% *v/v* of carrier concentration, it remained constant for a higher concentration of the carrier. This behavior is due to that at these higher concentrations of the ionic liquid, mass transfer control is shifted to the feed phase, thus, an increase in the ionic liquid concentration does not influence the mass transfer significantly.

The influence of the Au(III) concentration in the feed phase on the overall mass transfer coefficient is showed in Table 2. As it can be observed from this Table, gold transport during the elapsed time (3 h) changed when the metal concentration varies between 0.01 and 0.15 g/L; these results are not in accordance with constant values of the distribution coefficient in the same range of gold concentrations when determined performing conventional liquid-liquid extraction. Accordingly, with the above, the overall mass transfer coefficient decreased from 3.2 × 10^−3^ cm/s to 1.4 × 10^−3^ cm/s when the concentration of Au(III) in the feed phase was increased from 0.01 to 0.15 g/L, and these results could be explained due to: (i) the organic solution immobilized within the support pores get saturated with gold complex on increasing the metal concentration in the feed solution, and (ii) the as-formed organic complex diffuses slowly into the bulk of the organic solution which resulted in decreased the mass transfer in the organic solution.

**Figure 5:**
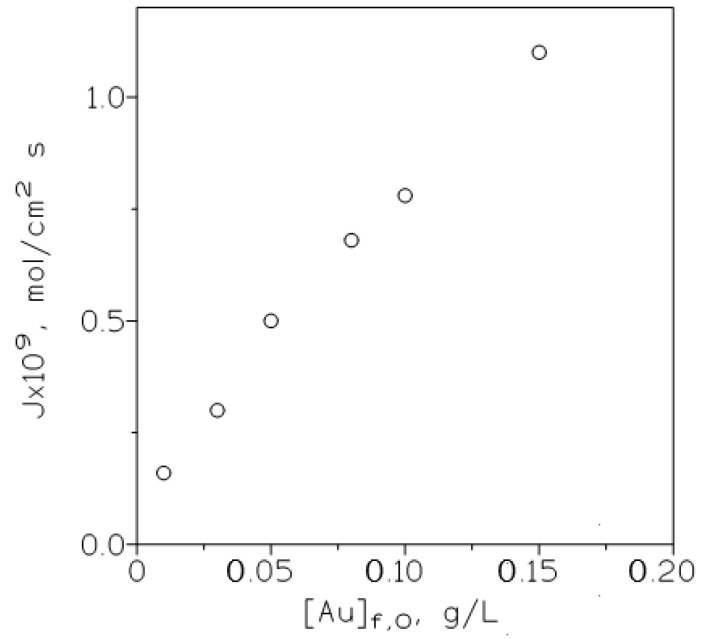
Variation of the metal flux (J) with the initial gold concentration in the feed phase. Feed phase: various gold concentrations in 3 M HCl. Other experimental conditions as in Table 2.

The influence of the initial gold(III) concentration in the feed phase on the metal flux was also considered by the use of the next relationship:

(9) $$J=K_{\mathrm{Au}}{\left[\mathrm{Au}\right]}_{f,0}$$

and plotting (Figure 5) J versus [Au]_f,0_. It is shown that as expected from the above equation, the flux increased continuously in the range of gold(III) concentrations investigated in this work, also this result indicated that the membrane is not saturated because gold is effectively stripped from the membrane to the receiving phase.

Since the resistance due to the receiving phase is considered as negligible, the metal flux through the membrane support can be described by applying Fick´s first diffusion law to the feed phase diffusion layer and to the membrane [33], thus:

(10) $$J_{f}=\Delta_{f}^{-1}\left({\left[\mathrm{Au}\right]}_{f,0}-{\left[\mathrm{Au}\right]}_{f,i}\right)$$

(11) $$J_{m}=\Delta_{m}^{-1}{\left[R_{3}{\mathrm{NH}}^{+}\mathrm{Au}{\mathrm{Cl}}_{4}^{-}\right]}_{f,i}$$

where [Au]_f,0_ is the initial gold concentration in the feed phase, and [Au]_f,i_ and [R_3_NH^+^AuCl_4_^−^]_f,i_ are the concentrations of the respective species in the feed/membrane interface. 

Considering that the extraction reaction (Equation (6)) is assumed to be fast in relation to the diffusion rate, local equilibria at the interface are related by K_ext_ (Equation (7)), then, at steady state J = J_f_ = J_m_ and combining Equations (6), (10) and (11), a final expression for the flux is obtained:

(12) $$J=\frac{\left(K_{\mathrm{ext}}{\left[R_{3}{\mathrm{NH}}^{+}{\mathrm{Cl}}^{-}\right]}_{m}{\left[{\mathrm{Cl}}^{-}\right]}_{f}^{-1}\right){\left[\mathrm{Au}\right]}_{f,0}}{\Delta_{m}+\Delta_{f}\left(K_{\mathrm{ext}}{\left[R_{3}{\mathrm{NH}}^{+}{\mathrm{Cl}}^{-}\right]}_{m}{\left[{\mathrm{Cl}}^{-}\right]}_{f}^{-1}\right)}$$

where Δ_f_ and Δ_m_ are the transport resistances due to diffusion across the feed phase boundary layer and the membrane, respectively, whereas [Au]_f,0_ is the initial metal concentration in the feed phase.

From Equation (12), the overall mass transfer coefficient is:

(13) $$K_{\mathrm{Au}}=\frac{K_{\mathrm{ext}}{\left[R_{3}{\mathrm{NH}}^{+}{\mathrm{Cl}}^{-}\right]}_{m}{\left[{\mathrm{Cl}}^{-}\right]}_{f}^{-1}}{\Delta_{m}+\Delta_{f}\left(K_{\mathrm{ext}}{\left[R_{3}{\mathrm{NH}}^{+}{\mathrm{Cl}}^{-}\right]}_{m}{\left[{\mathrm{Cl}}^{-}\right]}_{f}^{-1}\right)}$$

This equation contained both the equilibrium and diffusion parameters involved in the transport of Au(III) across a supported liquid membrane in which the carrier dissolved in toluene is immobilized. The values of the resistances to the mass transfer can be determined by the next relationship:

(14) $$\frac{1}{K_{\mathrm{Au}}}=\Delta_{f}+\Delta_{m}\frac{1}{K_{\mathrm{ext}}{\left[R_{3}{\mathrm{NH}}^{+}{\mathrm{Cl}}^{-}\right]}_{m}{\left[{\mathrm{Cl}}^{-}\right]}_{f}^{-1}}$$

A plot of 1/K_Au_ versus 1/K_ext_[R_3_NH^+^Cl^−^]_m_[Cl^−^]_f_^−1^, for different carrier concentrations and 3 M HCl, resulted in a straight line (r^2^ = 0.911) with intercept Δ_f_ and slope Δ_m_. Thus, the values of the transport resistances due to diffusion by the aqueous feed boundary layer and the membrane estimated from the proposed model are 307 and 9.5 × 10^6^ s/cm, respectively. The mass transfer coefficient in the feed phase is Δ_f_^−1^ = 3.3 × 10^−3^ cm/s, and assuming an overall diffusion coefficient (D_f_) of the gold species in the feed solution as 1 × 10^−5^ cm^2^/ s, and:

(15) $$d_{f}=\frac{D_{f}}{\Delta_{f}^{-1}}$$

the thickness of the aqueous boundary layer (d_f_) was estimated as 3.0 × 10^−3^cm, which was in agreement with other values derived from other gold(III)-HCl-transport systems [23,24,25,34,35,36,37]. 

The diffusion coefficient in the organic solution:

(16) $$D_{m}=\frac{d_{m}}{\Delta_{m}}$$

was estimated as 1.3 × 10^−3^ cm^2^/s, considering Δ_m_ = 9.5 × 10^6^ s/cm and the thickness of the membrane support, d_m_, as 12.5 × 10^−3^ cm.

The diffusion coefficient of the gold(III)-ionic liquid species in the bulk organic phase is estimated using the next relationship [38]:

(17) $$D_{b,m}=D_{m}\frac{\tau^{2}}{\varepsilon }$$

where τ is the membrane tortuosity (1.67) and ε is the support porosity (75%), thus, D_b,m_ is estimated as 4.8 × 10^−3^ cm^2^/s.

The diffusion coefficient in the bulk organic phase presented a greater value than the diffusion coefficient being this attributable to the diffusional resistance caused by the support thickness separating the feed and receiving solutions.

Considering that the ionic liquid concentration in the membrane support is constant, the apparent diffusion coefficient for gold (III) can be calculated as:

(18) $$D_{m}^{a}=\frac{{\mathrm{Jd}}_{m}}{\left[R_{3}{\mathrm{NH}}^{+}{\mathrm{Cl}}^{-}\right]}$$

using a 10% *v/v* ionic liquid concentration (2.1 × 10^−1^ M), and being d_m_ 12.5 × 10^−3^ cm, this apparent diffusion coefficient has the value of 9.5 × 10^−5^ cm^2^/s.

**Figure 6:**
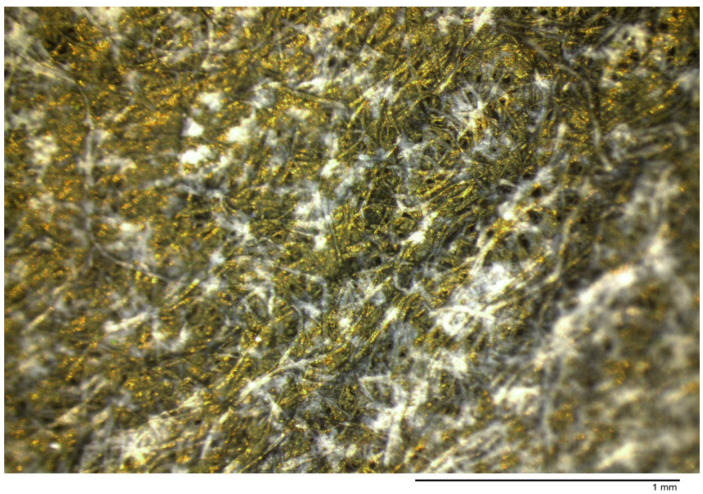
Gold nanoparticles observed under the optical microscope.

**Figure 7:**
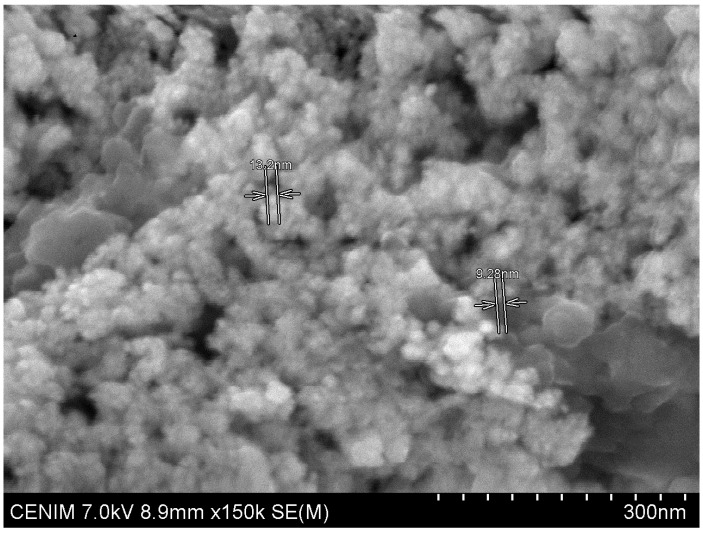
SEM image of the precipitated gold nanoparticles. Marked particles size: 13.2 nm (**upper**), 9.28 nm (**lower**).

**Figure 8:**
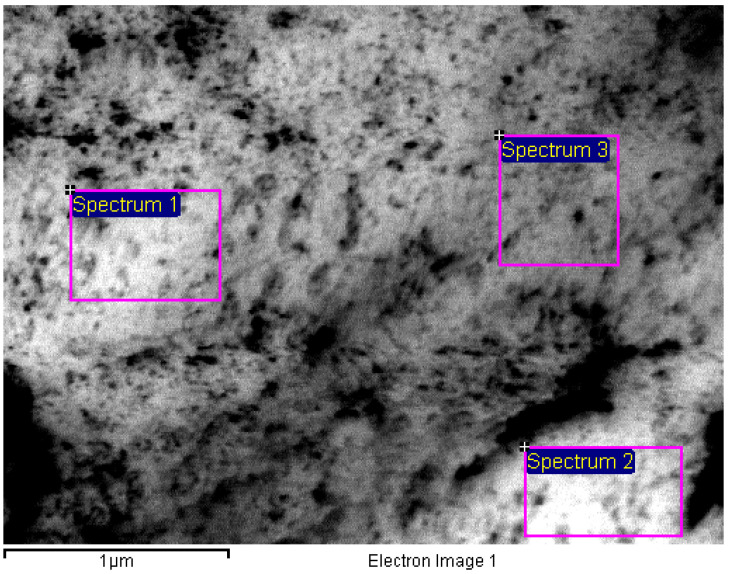
SEM image for Energy Dispersive x-ray Spectroscopy microanalysis.

Gold-bearing solutions collected from the receiving phases were precipitated by a 0.1 M sodium borohydride solution, the dry solid formed has the appearance showed in Figure 6, it can be recognized the bright colour of metallic gold, which precipitates in the form of nanoparticles, this was corroborated by SEM, as it is shown in Figure 7 and Figure 8 and Table 3.

The reduction of gold(III) to zero valent gold responded to the formation of H_2_ in the hydrolysis of sodium borohydride and the subsequent next reaction:

(19) $$2\mathrm{Au}{\left(\mathrm{SCN}\right)}_{4}^{-}+3H_{2}\to 2{\mathrm{Au}}^{0}+6N^{+}+8{\mathrm{SCN}}^{-}$$

## 4. Conclusions

The reaction between the tertiary amine A327 and hydrochloric acid generated the ionic liquid A327H^+^Cl^−^, this ionic liquid dissolved in toluene extracted gold(III) from 3 M HCl solution. Gold(III) extraction by this ionic liquid is attributed to an anion exchange reaction, with the formation of A327H^+^AuCl_4_^−^ species in the organic phase. The extraction system has been implemented in a supported liquid membrane process in which, and under the present experimental conditions, metal flux increased with the increase of the initial metal concentration in the feed phase. Metal permeation is dependent on ionic liquid concentration, though from a carrier concentration of 5% v/v in toluene, a limiting permeability value is reached and under this condition the transport process is controlled by diffusion in the feed phase boundary layer; at carrier concentrations lower than 5% *v/v* membrane diffusion controlled the overall gold(III) transport. Mass transfer coefficients in the feed and membrane phases are found to be 3.3 × 10^−3^ and 1.1 × 10^−7^ cm/s, respectively. From the receiving solutions, gold(III) can be precipitated as gold nanoparticles.

## Figures and Tables

**Table 1:** Influence of the stirring speed on transport of Au(III) as a function of the overall mass transfer coefficient (K_Au_).

Stirring Speed, min^−1^	K_Au_ × 10^3^, cm/s
600	2.6
800	2.7
900	2.9
1000	3.2
1200	3.0
1500	3.1
1800	3.2

**Table 2:** Mass transfer coefficients for transport of Au(III) from 3 M HCl medium as a function of initial Au(III) concentration in the feed phase.

[Au]_f,0_, g/L	K_Au_ × 10^3^, cm/s	ª % Transport
0.01	3.2	86
0.03	2.0	78
0.05	2.0	70
0.08	1.7	67
0.10	1.5	61
0.15	1.4	57

Feed phase stirring speed: 1000 min^−1^. Membrane phase: 10% *v/v* carrier in toluene supported on GVHP4700. Receiving phase: 0.25 M NaSCN. Stirring speed: 500 min^−1^. Temperature: 20 °C. ª after 3 h and calculated as ([Au]_f,0_ − [Au]_f,t_) × 100/[Au]_f,0._

**Table 3:** Energy Dispersive x-ray Spectroscopy microanalysis microanalysis of the areas shown in Figure 8.

Spectrum	Au (wt %)
1	100.00
2	100.00
3	100.00
Mean	100.00
Standard deviation	0.00
